# Epigenetic regulation of interleukin-8, an inflammatory chemokine, in osteoarthritis

**DOI:** 10.1016/j.joca.2015.02.168

**Published:** 2015-11

**Authors:** A. Takahashi, M.C. de Andrés, K. Hashimoto, E. Itoi, R.O.C. Oreffo

**Affiliations:** † Bone and Joint Research Group, Centre for Human Development Stem Cells and Regeneration, Institute of Developmental Science, University of Southampton Medical School, Southampton, UK; ‡ Department of Orthopaedic Surgery, Tohoku University School of Medicine, Sendai, Japan

**Keywords:** Interleukin-8, Chemokine, DNA methylation, Epigenetics, Inflammation, Osteoarthritis

## Abstract

**Objective:**

To determine whether altered *IL8* methylation status is associated with increased expression of *IL8* in human osteoarthritic (OA) chondrocytes.

**Methods:**

*IL8* expression levels and the percentage CpG methylation in human chondrocytes were quantified by qRT-PCR and pyrosequencing in OA patients and in non-OA osteoporotic controls. The effect of CpG methylation on *IL8* promoter activity was determined using a CpG-free vector; co-transfections with expression vectors encoding nuclear factor-kappa B (NF-κB), AP-1 and C/EBP were subsequently undertaken to analyse for *IL8* promoter activity in response to changes in methylation status.

**Results:**

*IL8* expression in OA patients was 37-fold higher than in osteoporotic controls. Three CpG sites in the *IL8* promoter were significantly demethylated in OA patients. Multiple regression analysis revealed that the degree of methylation of the CpG site located at −116-bp was the strongest predictor of *IL8* expression. *In vitro* DNA methylation was noted to decrease *IL8* promoter basal activity. Furthermore, NF-κB, AP-1 and C/EBP strongly enhanced *IL8* promoter activity whilst DNA methylation inhibited the effects of these three transcription factors.

**Conclusions:**

The present study demonstrates the key role of DNA methylation status on the expression of *IL8* in human chondrocytes. We demonstrate a quantitative relationship between percentage methylation and gene expression within clinical samples. These studies provide direct evidence linking the activation of *IL8*, DNA demethylation and the induction of the OA process with important therapeutic implications therein for patients with this debilitating disease.

## Introduction

Osteoarthritis (OA) remains, currently, the most frequent cause of pain, deformity and dysfunction in the elderly population^1^, ^2^. Although the pathogenesis of OA is far from clear, involvement of inflammation in the development and progression of OA has been implicated^3^, ^4^. Indeed, epidemiological studies indicate a significant association between OA disease progression and the presence of inflammatory synovium^5^, ^6^. Furthermore, a notable finding of inflammation in the tissue is the recruitment of neutrophils from the blood to the affected site; a process mediated by chemokines, small 8–12 kD chemotactic proteins^7^.

Interleukin 8 (IL-8), also named CXCL-8, is an inflammatory chemokine present under pathological conditions. IL-8, produced by human OA chondrocytes, is an important mediator in the pathophysiology of OA^8^, ^9^, ^10^, ^11^ including promotion of a number of pathogenic processes such as; (1) release of matrix metalloproteinase-13 (MMP-13), (2) neutrophil accumulation and, (3) activation and leukocyte homing to the synovium^12^, ^13^, ^14^. Furthermore, IL-8 and other chemokines are known to induce chondrocyte hypertrophy and differentiation^9^, ^15^, ^16^. Pierzchala *et al.* reported that synovial fluid from OA patients displayed significantly increased levels of IL-8 compared to controls^17^.

**Fig. 1:**
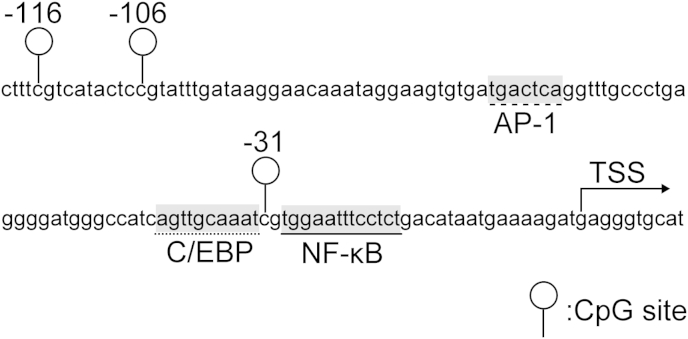
Diagrammatic representation of CpG sites within the proximal *IL8* promoter with CpG sites and location of transcription factors indicated.

Gene expression is regulated by epigenetic and non-epigenetic mechanisms. Epigenetics refers to heritable or stable, long-term changes in gene activity without changes in the DNA sequence. DNA methylation at CpG sites is a central epigenetic mechanisms conferring long-term regulation of set genes in contrast to regulation observed by histone modifications^18^, ^19^, ^20^. While DNA methylation of the so-called CpG islands have been predominantly examined, a few studies have shown that single or a few specific CpG sites can dominate the promoter activities of a particular gene^21^, ^22^, ^23^, ^24^. DNA methylation at CpG sites has been shown as a critical mediator in human OA chondrocytes for a number of key genes implicated in OA including *IL1B*, *MMP13, iNOS* and *COL9A1*^21^, ^24^, ^25^, ^26^. Furthermore, CpG methylation status can directly affect the binding of transcriptional factors resulting in altered transcriptional activity^24^. In previous studies, transcriptional regulation of *IL8* by nuclear factor-kappa B (NF-κB), activator protein-1 (AP-1) and CCAAT/enhancer-binding protein (C/EBP) has been reported in a number of tissues and cell types including colonic epithelial cells, ovarian cancer cells and myometrial cells^27^, ^28^, ^29^, ^30^, ^31^, ^32^. Critically, sequences spanning nucleotides −1 to −133 within the *IL8* proximal promoter were observed to be essential and sufficient for transcriptional regulation of the *IL8* gene. This sequence (−1 to −133) includes binding sites for NF-κB, AP-1 and C/EBP^28^, ^30^, ^33^. In addition, within *IL8*, the 1000-bp of the proximal promoter region contains only three CpG sites all located close to transcriptional binding sites (Fig. 1). However, the methylation status of these CpG sites and subsequent involvement in the regulation of *IL8* regulation remains, to date, unknown. The current study set out to examine whether the increased expression of *IL8* in human OA is a consequence of epigenetic regulation, specifically DNA hypomethylation.

## Materials and methods

### Chondrocyte isolation

Human articular cartilage was obtained from the femoral heads of patients undergoing hemiarthroplasty following a fracture of the neck of femur (#NOF) or after total hip arthroplasty for OA (OARSI score for OA grade^34^ in all OA patients was 3–4). Samples were derived with full patient consent and prior approval of the local Institutional Review Board. Given patients with a #NOF are likely to be suffering from osteoporosis, and the accepted inverse relation between OA and osteoporosis [17], cartilage from these patients served as control samples^35^. Cartilage tissue was dissected within 6 h of surgery from OA and non-OA samples and primary chondrocytes isolated as previously detailed in Refs. 26, 36, 37. Briefly, non-OA/healthy chondrocytes were isolated from the cartilage deep zone of patients with #NOF, whereas cartilage pieces adjacent to weight-bearing areas of OA femoral heads (lacking surface zones) were harvested for OA chondrocytes. Cartilage samples were dissected and cut into small fragments and digested with 10% trypsin (Lonza) in PBS for 30 min, followed by sequential digestion using 1 mg/ml of hyaluronidase (Sigma–Aldrich) in PBS for 15 min, and in 10 mg/ml of collagenase B (Roche Applied Science) in DMEM/F12 medium (Life Technologies) for 12–15 h at 37°C. Isolated chondrocytes from 15 #NOF samples (controls, 5 men and 10 women with a mean ± SD age of 84.5 ± 5.3) and 15 OA samples (OA, seven men and eight women with a mean ± SD age of 66.7 ± 12.5) were directly used for extraction of genomic DNA and total RNA. The chondrocytes from seven #NOF patients were cultured for culture experiments.

### Chondrocyte culture

Following cell isolation, non-OA chondrocytes were divided into three groups: (1) control culture, (2) IL-1β culture, and (3) 5-aza culture. Chondrocytes were cultured at a density of 2–4 × 10^5^ cells/25-cm^2^ flask in 5 ml of DMEM/F12 supplemented with 5% fetal calf serum (FCS; Invitrogen), 1% insulin–transferrin–selenium (Sigma–Aldrich), 100 units/ml of penicillin and 100 μg/ml of streptomycin (Lonza), and 100 μg/ml of ascorbic acid (Sigma–Aldrich) in the atmosphere of 5% CO_2_ at 37°C. IL-1β (10 ng/ml) (Sigma–Aldrich) and oncostatin M (10 ng/ml) (Sigma–Aldrich) were added to cultures based on the observations these inflammatory cytokines are elevated in OA synovial fluid and known to induce the short-term induction of catabolic genes and to alter DNA methylation status following long-term stimulation^4^, ^26^, ^38^. The primary cultures were maintained for 5 weeks until cells reached confluence.

### DNA and RNA extraction and molecular analysis (qRT-PCR)

Total RNA and genomic DNA were extracted simultaneously from the harvested chondrocytes using AllPrep DNA/RNA Mini kit (Qiagen). RNA was immediately reverse transcribed with SuperScript VILO cDNA Synthesis Kit (Life Technologies). Relative quantification of gene expression was performed with an ABI Prism 7500 detection system (Applied Biosystems). The 20-μl reaction mixture was prepared in triplicate, containing 1 μl of complementary DNA, 10 μl of Power SYBR Green PCR Master Mix (Applied Biosystems), and 250 nM of each primer. Thermal cycler conditions included an initial activation step at 95°C for 10 min, followed by a two-step PCR program of 95°C for 15 s and 60°C for 60 s for 40 cycles. The 2^−ΔΔCt^ method was used for relative quantification of gene expression. Reactions were performed in triplicate and samples normalised against GAPDH gene expression as control. GAPDH primers were designed using Primer Express software (version 3.0; Applied Biosystems). *IL8* primers were obtained from the PrimerBank database^39^ (PrimerBank ID: 10834978a2) and the primers used for quantitative reverse transcription PCR (qRT-PCR) are illustrated in Table I.

### Bisulfite pyrosequencing

Genomic DNA extracted from each sample was treated with sodium bisulfite to convert unmethylated cytosine in CpG sites to uracil using the EZ DNA Methylation-Gold Kit (Zymo Research Corporation). Following bisulfite treatment, PCR was performed with Premium PCR Supermix High Fidelity (Invitrogen). The percentage DNA methylation in the *IL8* promoter was quantified using PyroMark MD (Qiagen). All primers were designed with Pyrosequencing Assay Design Software (Qiagen) (Table I).

### Plasmid constructions

The *IL8* promoter constructs were generated by PCR amplification using genomic DNA from human articular chondrocytes as a template. The following PCR primers were used: 5′-ATAGGATCCGCCTTGCTCCAACTGCCTTT-3′ (forward) and 5′-AATCCATGGTGGTTTCTTCCTGGCTCTTGT-3′ (reverse). Underlined letters indicate BamHI and NcoI recognition sequences, respectively. The resultant PCR products were digested with BamHI and NcoI (Thermo Scientific) and transferred into the multiple cloning site of a pCpGfree-Luc vector using Rapid DNA Ligation Kit (Thermo Scientific). The vector lacks CpG sites within the whole vector sequence and was generated as detailed by Klug and Rehli^40^. Point mutations at CpG sites were generated by converting CG to TG using QuickChange II Site-Directed Mutagenesis Kit (Agilent Technologies). Primers for mutagenesis were designed using QuikChange Primer Design (Agilent Technologies) (Table I). Promoter constructs with a mutation at CpG sites located at −31-bp, −106-bp and −116-bp from the transcription start site (TSS) were generated according to the manufacturer's instructions. Promoter constructs with two mutations at the CpG sites were produced by two-step mutagenesis. In total, six mutation patterns were generated (Fig. 6). The sequences of all constructs were confirmed by DNA sequencing using SmartSeq system (Eurofins Genomics).

### *In vitro* methylation, transfection and luciferase assay

Plasmids were methylated using CpG Methylase M.Sssl (New England Biolabs). Complete methylation was verified by plasmid DNA bisulfite modification and pyrosequencing with specific primers. The immortalized human chondrocytes, C28/I2, were seeded at a density of 30,000 cells per well in 24-well plates, cultured in DMEM/F12 overnight, and transfected with a mixture of 300 ng luciferase reporter vector and 1 ng pRL-TK Vector (Promega), using FuGENE HD *in vitro* Transfection Reagent (Promega). Transfected C28/I2 cells were cultured for 48 h prior to harvest. Cell lysates were assayed for firefly and renilla luciferase activity using a Dual-Luciferase Reporter Assay System on a Varioskan Flash (Thermo Scientific). Firefly luciferase activity of each transfection was normalized against renilla luciferase activity. Reactions were performed in duplicate, and each experiment was repeated at least three times.

The expression vectors for NF-κB (p50, p65, or p50/p65), AP-1(c-Fos, c-Jun, or c-Fos/c-Jun) and C/EBPβ were used (60 ng) for co-transfections. Blank expression vector pCMV4, pcDNA3.1 and pcDNA3.1(+) served as controls, respectively. Total DNA was normalized with empty vectors in the transfection mixture.

### Statistical analysis

Statistical analysis was performed using SPSS Statistics (version 21.0; IBM). Cartilage samples were obtained from individual subjects. The Mann–Whitney *U* test was used to compare gene expression, CpG percentage methylation and age between two groups. Female to male ratios were compared using chi-square test. Spearman's rank correlation coefficient was used to analyse the relationship between percentage methylation and *IL8* expression and multiple regression analysis using least-squares method was applied to determine the relationship between *IL8* expression and patients' background data. Kruskal–Wallis test and Newman–Kuels multiple comparisons test were used to analyse the luciferase reporter assays. *P* values less than 0.05 were considered significant.

## Results

### *IL8* proximal promoter CpG sites are demethylated in OA chondrocytes and correlate with higher *IL8* gene expression

**Fig. 2:**
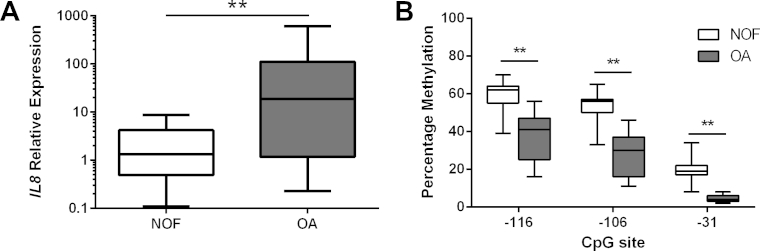
(A) Relative mRNA expression of *IL8* in non-cultured primary human chondrocytes obtained from patients with femoral neck fracture (NOF) and OA patients. mRNA was analyzed by quantitative RT-PCR and normalized against GAPDH. (B) Percentage methylation of the indicated CpG sites in the *IL8* proximal promoter in the same samples analysed by bisulfite pyrosequencing. *Y*-axis shows non-adjusted percentage methylation. Values are the mean ± SD of 15 independent samples from each group. **P < 0.01.

Initial studies centred on quantification of the CpG methylation status of the *IL8* proximal promoter in human primary chondrocytes isolated from articular cartilage obtained from non-OA (#NOF) donors (*n* = 15) and patients with OA (*n* = 15). *IL8* expression in OA chondrocytes was observed to be 37-fold higher than in #NOF controls [Fig. 2(A)]. Pyrosequencing analysis of the *IL8* promoter in the same subjects revealed that all three CpG sites in the promoter region were significantly demethylated in OA chondrocytes in contrast to #NOF chondrocytes. OA chondrocytes displayed a 22%, 26% and 15% statistically significant (*P* < 0.01) reduction in methylation status at the −116, −106 and −31 CpG sites, respectively [Fig. 2(A)].

**Fig. 3:**
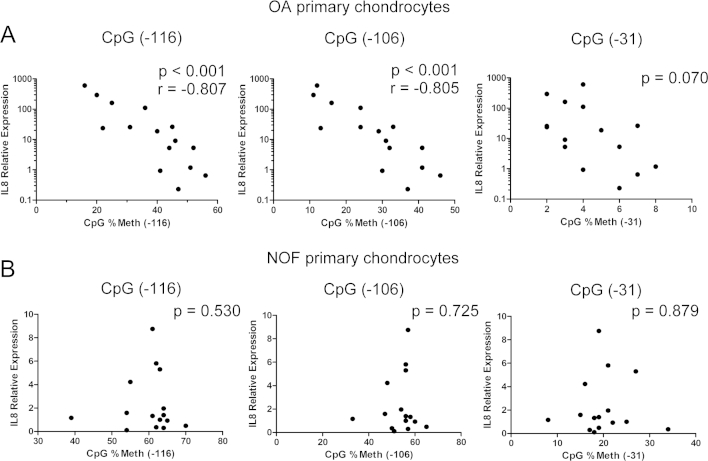
Results of Spearman's rank correlation coefficient comparing relative mRNA expression of IL8 and methylation status of the indicated CpG sites in the IL8 proximal promoter in OA chondrocytes (A) and controls (B).

A significant negative correlation was observed between *IL8* gene expression and the percentage methylation of the CpG sites located at −116-bp and −106-bp in OA chondrocytes [Fig. 3(A)]. The percentage methylation of the CpG site located at −31-bp displayed a correlation trend with *IL8* expression although this was not statistically significant (*P* = 0.069). In contrast, no correlation was observed between *IL8* expression and the percentage methylation in #NOF chondrocytes [Fig. 3(B)]. Importantly, multiple regression analysis revealed that the percentage methylation of the CpG site located at −116-bp was the strongest predictor of *IL8* expression (*P* < 0.01). Furthermore, advanced age and OA were also associated with higher *IL8* expression (Table II).

### Demethylation of CpG sites in the *IL8* promoter following long-term culture does not result in induction of *IL8* gene expression

**Fig. 4:**
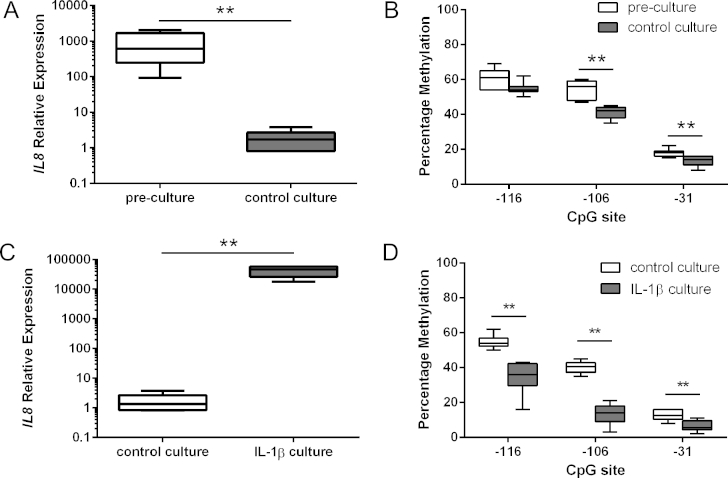
Relative mRNA expression of *IL8* was analyzed by quantitative RT-PCR and normalized against GAPDH in (A) pre-culture NOF chondrocytes and control culture chondrocytes, and (C) control culture and IL-1β culture. Percentage methylation of the indicated CpG sites in the *IL8* proximal promoter was analysed using bisulfite pyrosequencing in (B) pre-culture NOF chondrocytes and control culture chondrocytes, and (D) control culture and IL-1β culture. Values are the mean ± SD of seven independent experiments. *P < 0.05, **P < 0.01.

Given monolayer culture is known to affect the gene expression profile of chondrocytes^41^, *IL8* mRNA levels were analysed in pre-culture control chondrocytes compared with cultured chondrocytes over for 5 weeks. The culture of chondrocytes resulted in a significant loss of *IL8* expression [Fig. 4(A)]. CpG sites located at −106-bp and −31-bp of the *IL8* promoter in the cultured chondrocytes showed significant demethylation compared with pre-culture chondrocytes [Fig. 4(B)].

### Long-term exposure to IL-1β and oncostatin M results in enhanced expression of *IL8* and loss of DNA methylation

Healthy chondrocytes were cultured for 5 weeks in IL-1β/OSM. Long-term treatment with IL-1β/OSM induced a 24,000-fold increase in *IL8* expression compared to control cultures [Fig. 4(C)]. Pyrosequence analysis of the *IL8* promoter revealed that #NOF chondrocytes cultured using IL-1β/OSM displayed a 22%, 25% and 2.3% reduction in methylation status at the −116, −106 and −31 CpG sites, respectively, in comparison to control cultures [Fig. 4(D)].

### Methylation decreases IL8 promoter activity *in vitro*

**Fig. 5:**
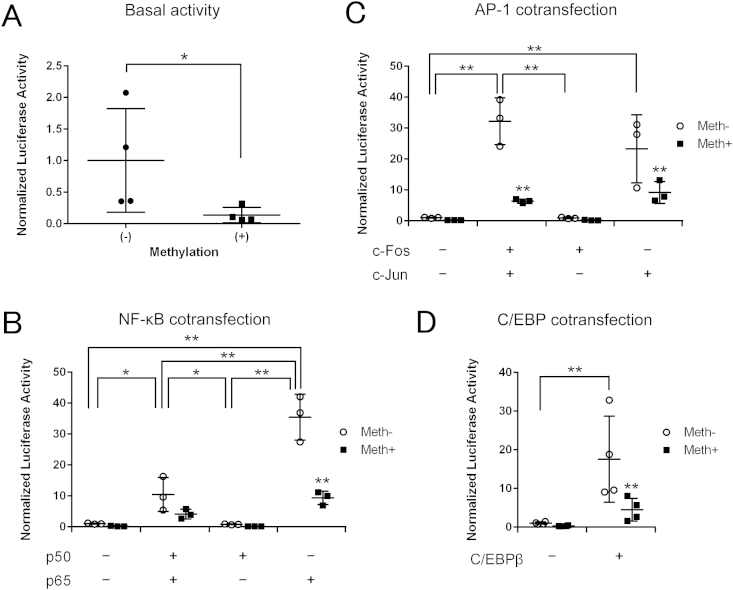
*IL8* promoter activity analysed by a luciferase assay in C28/I2 cell line transfected with non-methylated or methylated CpG-free vector containing wild-type *IL8* promoter construct. (A) Basal activities without treatment. (B) Co-transfection with the empty control vector (pCMV4) or with the NF-κB p50 subunit expression vector, p65 subunit expression vector or both. (C) Co-transfection with the empty control vector (pcDNA3.1) or with the AP-1 c-Fos subunit expression vector, c-Jun subunit expression vector or both. (D) Co-transfection with the empty control vector (pcDNA3.1(+)) or with the C/EBPβ expression vector. Values are the mean ± SD of three independent experiments (B and C) or four (A and D). *P < 0.05, **P < 0.01.

To determine the effects of DNA methylation on *IL8* promoter activity, dual-luciferase reporter assays were performed. The C28/I2 chondrocyte cell line was transfected with the wild type *IL8* promoter construct using a CpG free vector and pRL-TK vector as an internal control. The luciferase assay was performed 48 h after transfection. Methylation treatment significantly decreased the activities of the promoter constructs by seven fold [Fig. 5(A)].

### NF-κB, AP-1 and C/EBPβ mediate *IL8* transactivation in human chondrocytes and CpG methylation impairs *IL8* promoter transactivation

To determine the effects of DNA methylation and the transcription factors NF-κB, AP-1 and C/EBP and on *IL8* promoter activity, the expression vector encoding each transcription factor and the control empty vectors were co-transfected with wild type *IL8* promoter construct using a CpG free vector. *IL8* promoter activity was significantly enhanced (35-fold) with the NF-κB p65 subunit whilst, in contrast, DNA methylation suppressed the effect of NF-κB on *IL8* activity [Fig. 5(B)]. The AP-1 c-Jun subunit significantly transactivated *IL8* by 23-fold and the enhanced activity was higher with overexpression of c-Fos and c-Jun combined (32-fold) [Fig. 5(C)]. C/EBPβ significantly enhanced *IL8*-driven reporter activity 17-fold while DNA methylation significantly reduced the effect of C/EBPβ [Fig. 5(D)].

### Mutations at three CpG sites proximal to the TSS increase *IL8* promoter basal activity

**Fig. 6:**
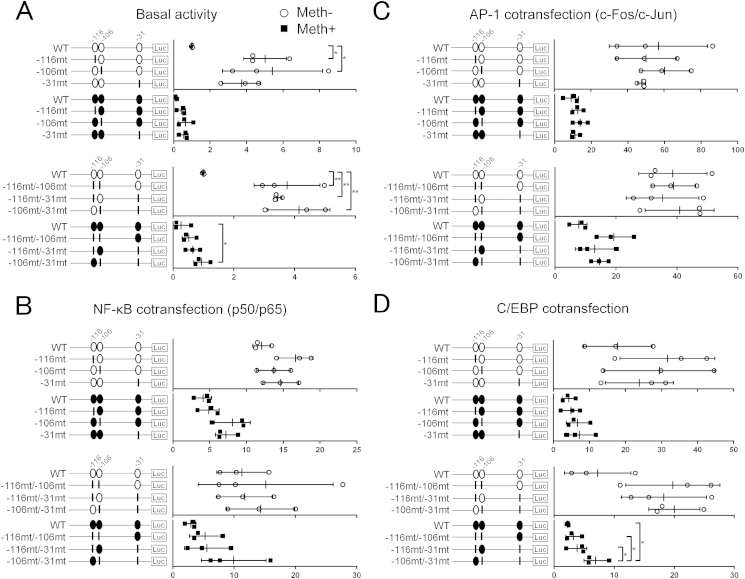
Assessment of promoter activity of methylated or non-methylated *IL8* promoter constructs containing different mutations analysed by luciferase assay. Point mutations (CG to TG) were created at CpG sites located at −31-bp, −106-bp, −116-bp, −116-bp and −106-bp, −116-bp and −31-bp, or −106-bp and −31-bp. (A) Basal *IL8* promoter activity in C28/I2 cell line transfected with methylated or non-methylated CpG-free vector containing wild-type or mutated *IL8* promoter constructs. (B) Co-transfection with the NF-κB p50 and p65 subunit expression vectors. (C) Co-transfection with the AP-1 c-Fos and c-Jun subunit expression vectors. (D) Co-transfection with the C/EBPβ expression vector. Values are the mean ± SD of three independent experiments. *P < 0.05, **P < 0.01.

To determine the CpG sites critical for *IL8* promoter activity, we compared *IL8* wild type promoter construct activity using a CpG free vector against six vectors containing mutations at different CpG sites [Fig. 6(A)]. Point mutations created on any single CpG site or two CpG sites resulted in a significant increase in *IL8* promoter activity by 3.4–5.4 fold [Fig. 6(A)]. Furthermore, non-methylated **−**106/**−**31 mutant constructs showed a significant increase in promoter activity (2.7–4.5 fold).

### NF-κB, AP-1 and C/EBPβ mediate *IL8* transactivation in cooperation with specific CpG sites within the proximal promoter

Sequences spanning nucleotides −1 to −133 within the *IL8* proximal promoter were observed to be essential and sufficient for transcriptional regulation of the *IL8* gene^30^. This sequence includes binding sites for NF-κB, AP-1 and C/EBP, and the three CpG sites [Fig. 1].

To evaluate the role of each CpG site for transcription factor-mediated *IL8* transactivation, the *IL8* wild type promoter construct and the six vectors with point mutations were co-transfected with the expression vectors encoding NF-κB, AP-1 and C/EBPβ. NF-κB overexpression increased the activity of wild type and mutated non-methylated promoter constructs. Point mutations created at **−**31-bp CpG or **−**106-bp CpG or both displayed a trend for increased NF-κB-driven *IL8* promoter transactivation in methylated plasmids [Fig. 6(B)]. In contrast, following AP-1 overexpression, methylated −116/−106 mutant constructs were observed to enhance *IL8* promoter activity [Fig. 6(C)]. Promoter activity pattern under C/EBP overexpression was similar to that observed following NF-κB overexpression. Non-methylated **−**106/**−**31 mutant constructs showed a significant increase in the promoter activity (2.8–3.1 fold) [Fig. 6(D)].

## Discussion

The current study demonstrates that the increased expression of *IL8* in human OA chondrocytes is regulated by DNA demethylation in cooperation with transcription factors. We show for the first time that the percentage methylation of specific CpG sites correlates with *IL8* gene expression level in clinical OA samples. Furthermore, long-term stimulation with IL-1β, a key pro-inflammatory cytokine involved in the pathophysiology of OA, resulted in the marked induction of *IL8* with decreased CpG methylation in the *IL8* promoter in human chondrocytes. Furthermore, 5-aza-dC treatment induced hypomethylation at the CpG site located at −116-bp in the IL8 promoter and enhanced IL8 expression (data not shown). Moreover, dual-luciferase reporter assays using a CpG free vector revealed that methylation treatment significantly decreased the activity of the *IL8* promoter constructs, thus the methylation status of CpG sites is one of the key transcriptional regulators of IL-8 in human chondrocytes. Interestingly, methylation status of the CpG sites did not correlate with *IL8* expression level in #NOF chondrocytes. Furthermore, a significant DNA demethylation of the *IL8* promoter in cultured chondrocytes did not result in increased *IL8* gene expression compared with pre-culture chondrocytes. These results suggest that methylation status alone was not sufficient to regulate *IL8* transcription. However, in pathological situations such as OA, methylation status of specific CpG sites appears crucial to the regulation of *IL8* expression. Critically, multiple regression analysis indicated that the methylation status of the CpG sites located at −116-bp from the TSS provided a strong association with *IL8* expression. Interestingly, this CpG is the most distal to the transcriptional binding sites and, thus should be the least affected. However, to date, there is a paucity of information concerning the three-dimensional configuration of the chromatin in this region in combination with transcription factors and indeed DNA methylation status and thus this remains an area for further study. Future studies with enhanced patient numbers including different grades of clinical OA, would be necessary to confirm and reveal the relationship of DNA methylation status with disease progression.

The primary functions of IL-8 are chemotaxis and angiogenesis and IL-8 has been shown to play an essential role in acute inflammation^7^, ^42^, ^43^. In general, OA is considered to be a “non-inflammatory arthritis”. However, growing evidence indicates the involvement of inflammation in the development and progression of OA^4^, ^5^. A recent methylome study revealed an enrichment of several pathways involved in inflammation including IL2, IL3, IL4 and IL6^44^. Furthermore, SOCS2 and CIS-1, inhibitors of cytokine signalling, have been shown to be suppressed in OA^45^. Enhanced expression of *IL8* in OA chondrocytes in the present study supports an association, in part, of OA with inflammation. Thus, disease progression and joint symptoms could potentially be modified by modulation and control of *IL8* expression. The current findings illustrating that DNA demethylation accounts for an increase in *IL8* expression in OA suggesting a potential target for OA modulation and warranting further (clinical and *in vivo*) examination.

Interestingly, *IL8* expression is typically, low or undetectable in normal non-inflammatory tissue. This is partly a result of transcriptional repression of the *IL8* promoter^30^. The *IL8* promoter contains a negative regulatory element (NRE) to which the NF-κB-repressing factor (NRF) binds. Reduction of cellular NRF by expressing NRF-antisense RNA results in spontaneous *IL8* gene expression^27^. Additionally, mutation of the NRE site results in loss of NRF binding and increased basal *IL8* expression^27^. The existence of a basal repression mechanism offers an explanation for the mutagenesis results observed in the current study. Point mutations created at the CpG sites in the *IL8* promoter could interrupt basal repression and result in increased promoter activity. In addition, the present studies indicate methylated *IL8* promoter constructs display low promoter activity indicating DNA methylation is an additionally basal repression mechanism of *IL8* expression.

The transcription factors, NFκB, CEBP, and AP-1, have all been implicated in *IL8* expression in a number of cell types^27^, ^28^, ^29^, ^30^, ^32^. NF-κB is a dimeric transcription factor composed of five different subunits^27^, ^30^, ^46^. We recently showed the NF-κB p65 subunit played a critical role in induction of *iNOS* in OA human chondrocytes in coordination with DNA demethylation of the enhancer elements^25^. NF-κB has also been shown to regulate the expression of a number of cytokines and chemokines, and several matrix degrading enzymes in OA pathogenesis^47^. A significant increase in *IL8* promoter activity by the NF-κB p65 subunit, AP-1 c-Jun subunit and C/EBPβ^27^, ^28^, ^29^, ^30^, ^31^, ^32^ was demonstrated in this study. Furthermore, the current study demonstrates that CpG methylation impairs *IL8* promoter transactivation by the transcription factors NF-κB, AP-1 and C/EBP. Interestingly, *IL8* transactivation by NF-κB and C/EBP were predominantly regulated by the CpG site located at −31-bp. In contrast, AP-1 was predominantly regulated by the CpG site located at −106-bp explained by the location of the CpG sites and the binding sites of the transcription factors [Fig. 1].

In conclusion, the present study demonstrates the key role of DNA methylation status on the expression of *IL8* in OA chondrocytes. This study demonstrates the quantitative relationship between percentage methylation of a CpG site and gene expression in clinical OA cartilage samples with evidence linking the activation of *IL-8*, DNA demethylation and, critically, induction of the OA process. These findings suggest, tentatively, a potential predictive marker, although additional *in vivo* and clinical studies are required before confirmation of this inflammatory chemokine, for pharmacological intervention in the treatment of OA and, potentially, other arthritic diseases.

## Author contributions

All authors were involved in drafting the article or revising it critically for important intellectual content, and all authors approved the final version to be published. In detail: AT—conception, design, acquisition of data, analysis and interpretation of data, drafting the manuscript; MCA—conception, design, acquisition of data, analysis and interpretation of data, critical revision of the manuscript; KH—conception, design, analysis and interpretation of data, critical revision of the manuscript; EI—analysis and interpretation of data, critical revision of the manuscript; ROCO—conception, design, analysis and interpretation of data, critical revision of the manuscript.

## Ethics approval

This study was approved by the Southampton & South West Hampshire Local Research Ethics Committee and informed consent was obtained from each patient.

## Role of the funding source

Funding from the Leverhulme Trust and Biotechnology and Biological Sciences Research Council (BB/G010579/1) to RO is gratefully acknowledged. The study sponsors had no direct involvement in the study, in writing of the manuscript or the decision to submit.

## Conflict of interest

The authors have no conflict of interest to declare.

## Figures and Tables

**Table I:** Primer sequences for (a) RT-PCR, (b) pyrosequencing, and (c) site directed mutagenesis

Name (length, bp)	Sequence (5′–3′)
(a)	
GAPDH (108)	F (CCAGGTGGTCTCCTCTGACTTC)
R (TCATACCAGGAAATGAGCTTGACA)

IL8 (112)	F (ACTGAGAGTGATTGAGAGTGGAC)
R (AACCCTCTGCACCCAGTTTTC)

(b)	
IL8-Pyro-1 (70)	F (AGGGGATGGGTTATTAGTTG)
R (ACTTATACACCCTCATCTTTTCATT)
S (GGATGGGTTATTAGTTGTA)

IL8-Pyro-2 (148)	F (GGTTTATTTTTTTAGGGTAAATTTGAGTTA)
R (ATTCACCAAATTATAAAACTTCAATATT)
S (ATTATATTTTTTATTTGTTTTTTATTAA)

(c)	
IL8-Mut1 (−116∗)†	F (AATTAAATTATTTTAAAGATCAAAGAAAACTTTtGTCATACTCCGTATTTGATAAGGAAC)
R (GTTCCTTATCAAATACGGAGTATGACaAAAGTTTTCTTTGATCTTTAAAATAATTTAATT)

IL8-Mut2 (−106∗)†	F (CAAAGAAAACTTTCGTCATACTCtGTATTTGATAAGGAACAAATAGG)
R (CCTATTTGTTCCTTATCAAATaCAGAGTATGACGAAAGTTTTCTTTG)

IL8-Mut3 (−31∗)†	F (GATGGGCCATCAGTTGCAAATtGTGGAATTTCCTCT)
R (AGAGGAAATTCCACaATTTGCAACTGATGGCCCATC)

IL8-Mut4 (−106∗)†	F (GATCAAAGAAAACTTTTGTCATACTCtGTATTTGATAAGGAACAAATAGGAAG)
R (CTTCCTATTTGTTCCTTATCAAATACaGAGTATGACAAAAGTTTTCTTTGATC)

F: forward; R: reverse; S: sequencing.

∗ Location of mutated CpG.

† Lowercase letters indicate a mutated base.

**Table II:** Factors associated with IL8 relative expression in human chondrocytes

	t value	*P* value	R^2^	*P* value
#NOF/OA [OA]	2.71	0.012		
Female/Male [Female]	0.81	0.427		
Age	2.42	0.024		
IL8 %Methylation (−31)	2.4	0.025		
IL8 %Methylation (−106)	2.59	0.016		
IL8 %Methylation (−116)	−3.64	0.001		

Total model			0.696	<0.0001

#NOF: a fracture of the neck of femur, OA: osteoarthritis.
